# Extensive ossifying fibroma of the mandible: segmental resection and free fibula flap reconstruction

**DOI:** 10.1093/jscr/rjag353

**Published:** 2026-05-09

**Authors:** Santosh Kumar Yadav, Srijana Dwa, Suresh Kumar Pradhan

**Affiliations:** Department of Oral and Maxillofacial Surgery, Bharatpur Hospital, Bharatpur-10, Chitwan 44200, Nepal; Department of Oral and Maxillofacial Surgery, Bharatpur Hospital, Bharatpur-10, Chitwan 44200, Nepal; Department of Plastic Surgery, Bharatpur Hospital, Bharatpur-10, Chitwan 44200, Nepal

**Keywords:** ossifying fibroma, mandible, free fibula flap, microvascular reconstruction, fibro-osseous lesion

## Abstract

Ossifying fibroma is a benign fibro-osseous neoplasm predominantly affecting young females, characterized by replacement of normal bone with fibrous tissue containing mineralized material. While smaller lesions can be managed conservatively, extensive lesions require resection to minimize recurrence. We report a 27-year-old female presenting with a large ossifying fibroma involving the right mandibular ramus, angle, and body. Clinical examination revealed facial asymmetry with firm, non-tender swelling, and buccal cortical expansion. Computed tomography demonstrated a well-defined, expansile lesion with mixed radiolucent-radiopaque internal structure. Segmental mandibulectomy with immediate free fibula flap reconstruction was performed. Microvascular anastomosis was completed under loupe magnification. Histopathology confirmed conventional ossifying fibroma. The patient was discharged on postoperative day 10 following suture removal, with excellent restoration of facial symmetry and minimal scarring. Unfortunately, the patient did not return for follow-up.

## Introduction

Ossifying fibroma is a benign fibro-osseous neoplasm originating from the periodontal ligament, characterized by replacement of normal bone with cellular fibrous tissue containing mineralized material [1, 2]. It predominantly affects females (63.9%) in the second and third decades of life, with 55%–93% occurring in the mandible [1, 3]. Radiographically, lesions demonstrate well-defined margins with variable radiolucent to radiopaque internal structure [1, 4]. While smaller lesions can be managed with curettage (recurrence rate 6.7%), extensive lesions require resection [1, 5]. The free fibula flap is the gold standard for mandibular reconstruction, with success rates of 97%–100% [6, 7].

We present a case of extensive ossifying fibroma involving the right mandibular ramus, angle, and body in a 27-year-old female, managed with segmental resection and immediate free fibula flap reconstruction. Treatment was provided pro bono as the patient could not afford expenses.

## Case report

**Figure 1:**
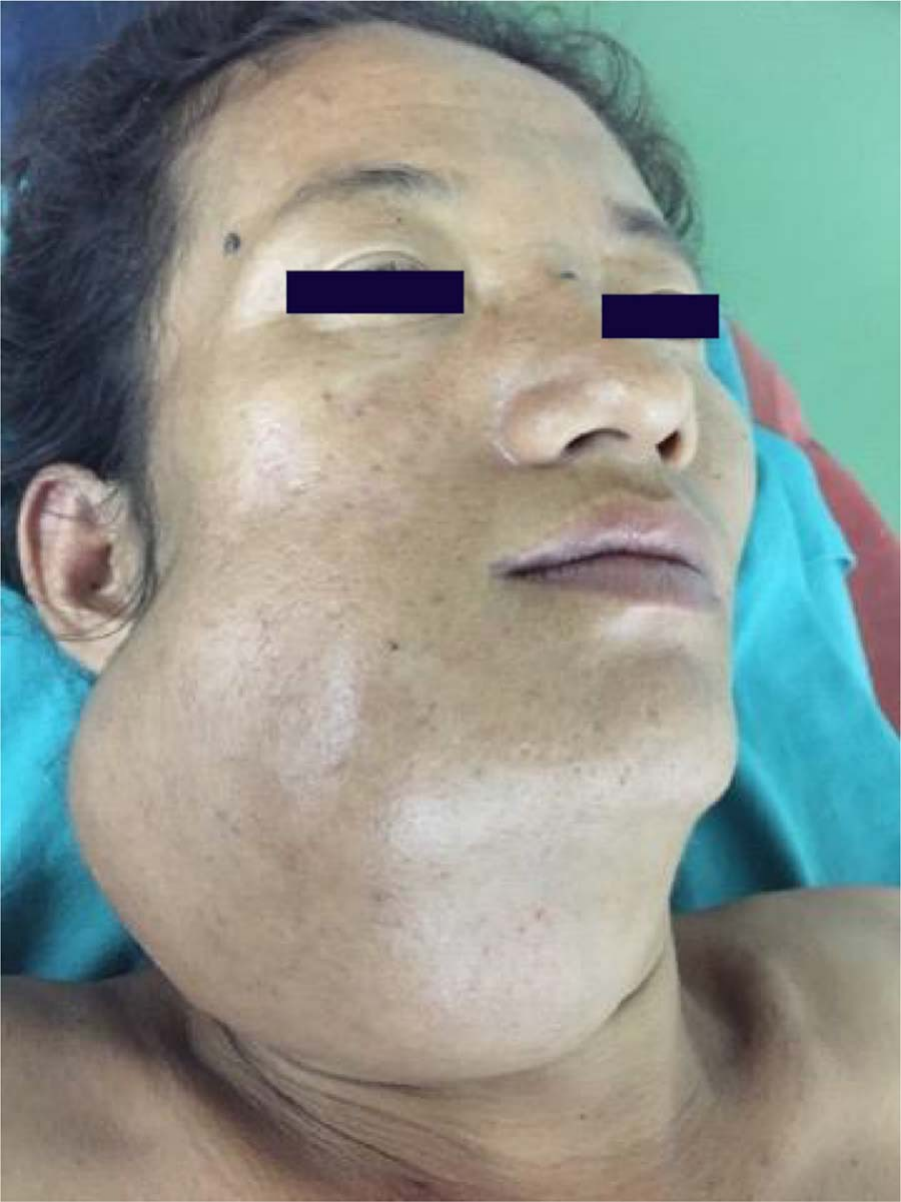
Preoperative clinical photograph. Extraoral view demonstrating diffuse right mandibular swelling with facial asymmetry.

A 27-year-old female from a remote area presented with progressive right mandibular swelling of 2 years' duration causing facial asymmetry (Fig. 1). The swelling was firm, non-tender, with normal overlying skin. Intraoral examination revealed buccal cortical expansion with tooth displacement and intact mucosa.

**Figure 2:**
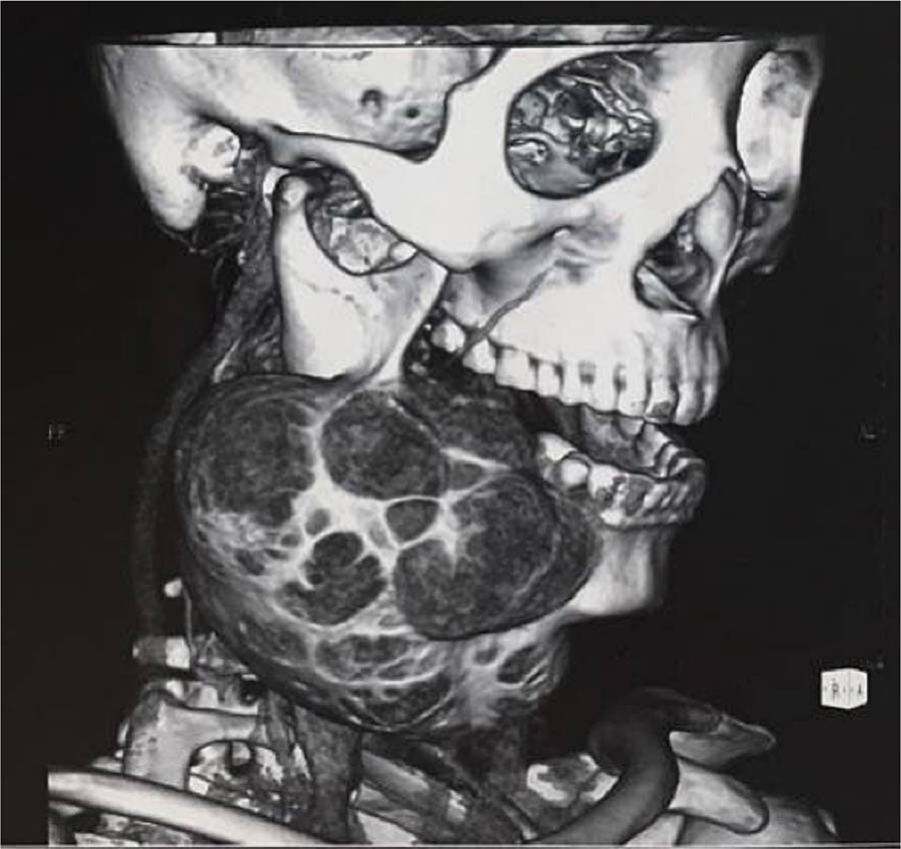
Preoperative CT. Three-dimensional reconstruction demonstrating extent of mandibular involvement.

Computed tomography (CT) demonstrated a well-defined, expansile lesion involving the right mandibular ramus, angle, and body with mixed radiolucent-radiopaque internal structure (Fig. 2). Cortical expansion with thinning was present without significant destruction. Incisional biopsy confirmed the diagnosis of ossifying fibroma.

**Figure 3:**
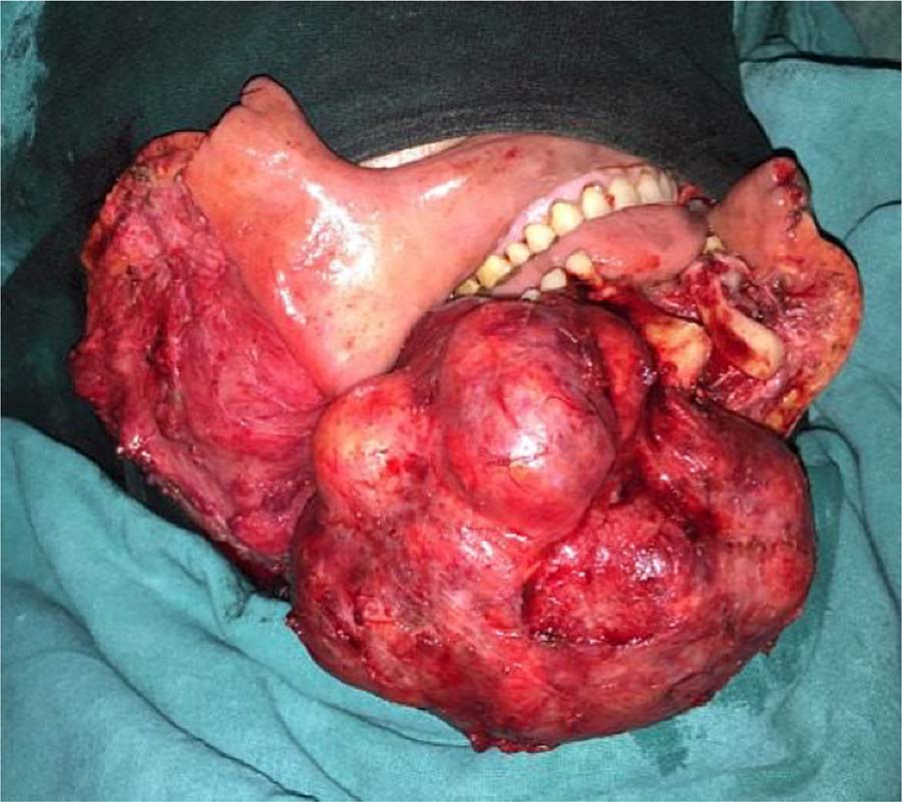
Intraoperative photograph. Resected mandibular segment with well-circumscribed lesion.

Segmental mandibulectomy with immediate free fibula flap reconstruction was planned. Under general anesthesia, a submandibular approach was used. The facial vessels were preserved as recipient vessels. Segmental resection was performed with 1 cm margins, and the resected specimen demonstrated a well-circumscribed lesion (Fig. 3). A template was modeled from the resected specimen to guide fibula contouring [8].

**Figure 4:**
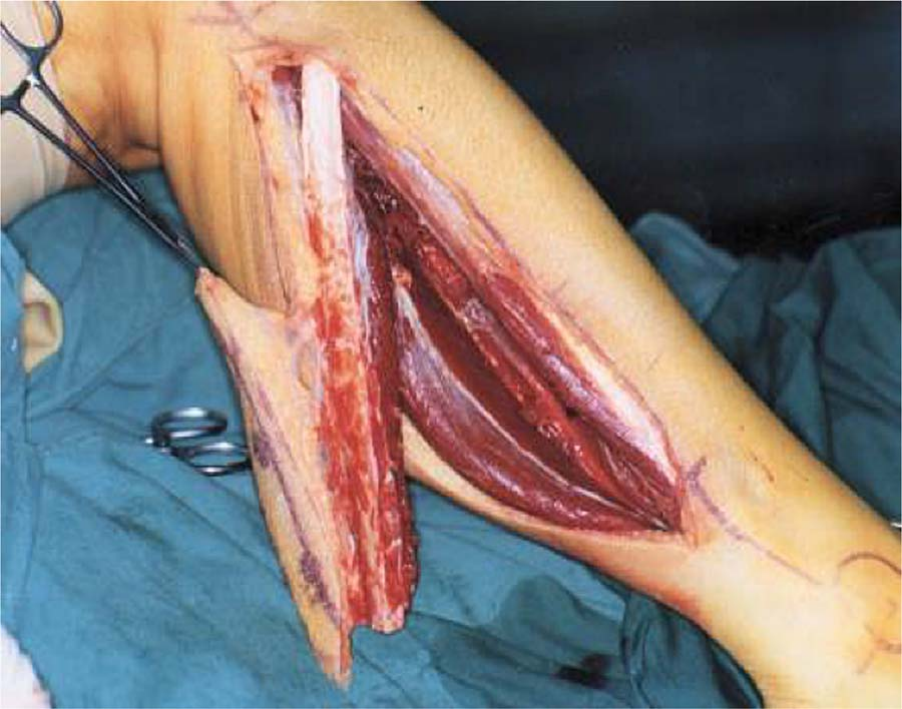
Harvested free fibula flap with vascular pedicle.

**Figure 5:**
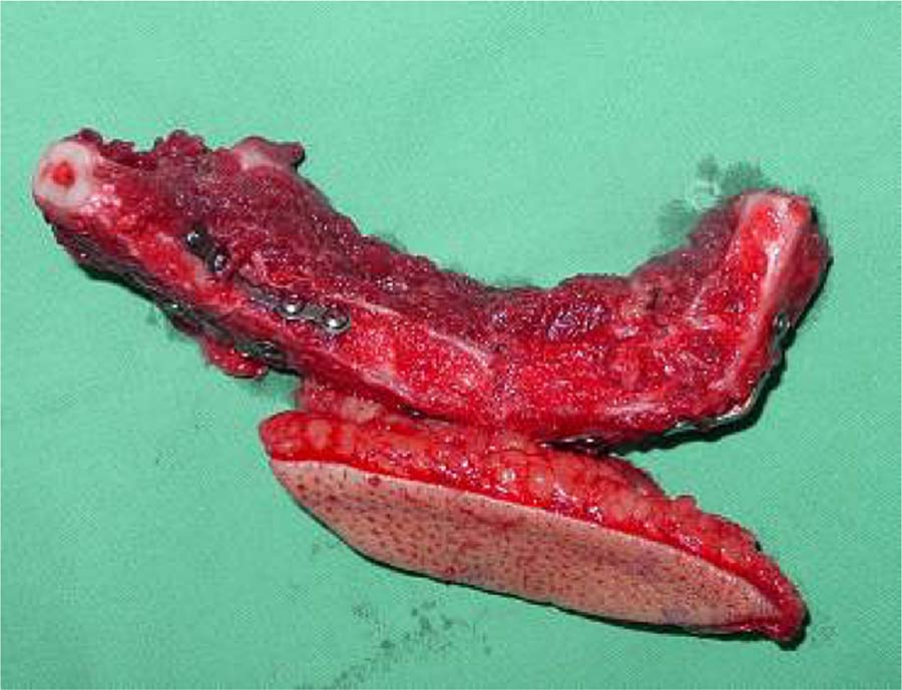
Fibula flap with a skin paddle, contoured with multiple osteotomies and miniplate fixation.

Simultaneously, a free fibula flap was harvested from the ipsilateral leg with the peroneal artery and venae comitantes as the vascular pedicle (Fig. 4). Approximately 12–14 cm of fibula was obtained, preserving 6–7 cm proximally and distally for joint stability [6, 9]. Multiple closing-wedge osteotomies were performed to recreate mandibular curvature, with miniplates maintaining segment alignment. The flap included a skin paddle for intraoral soft tissue coverage (Fig. 5) [8].

The contoured fibula was secured to native mandible using a titanium reconstruction plate. Microvascular anastomosis was performed under loupe magnification (×3.5), with the peroneal vessels anastomosed end-to-end to facial vessels using 9-0 nylon sutures. Loupe magnification provides comparable outcomes to the operating microscope for free tissue transfer [10, 11]. Flap perfusion was confirmed by skin paddle color, capillary refill, and Doppler signal. The donor site was closed primarily.

**Figure 6:**
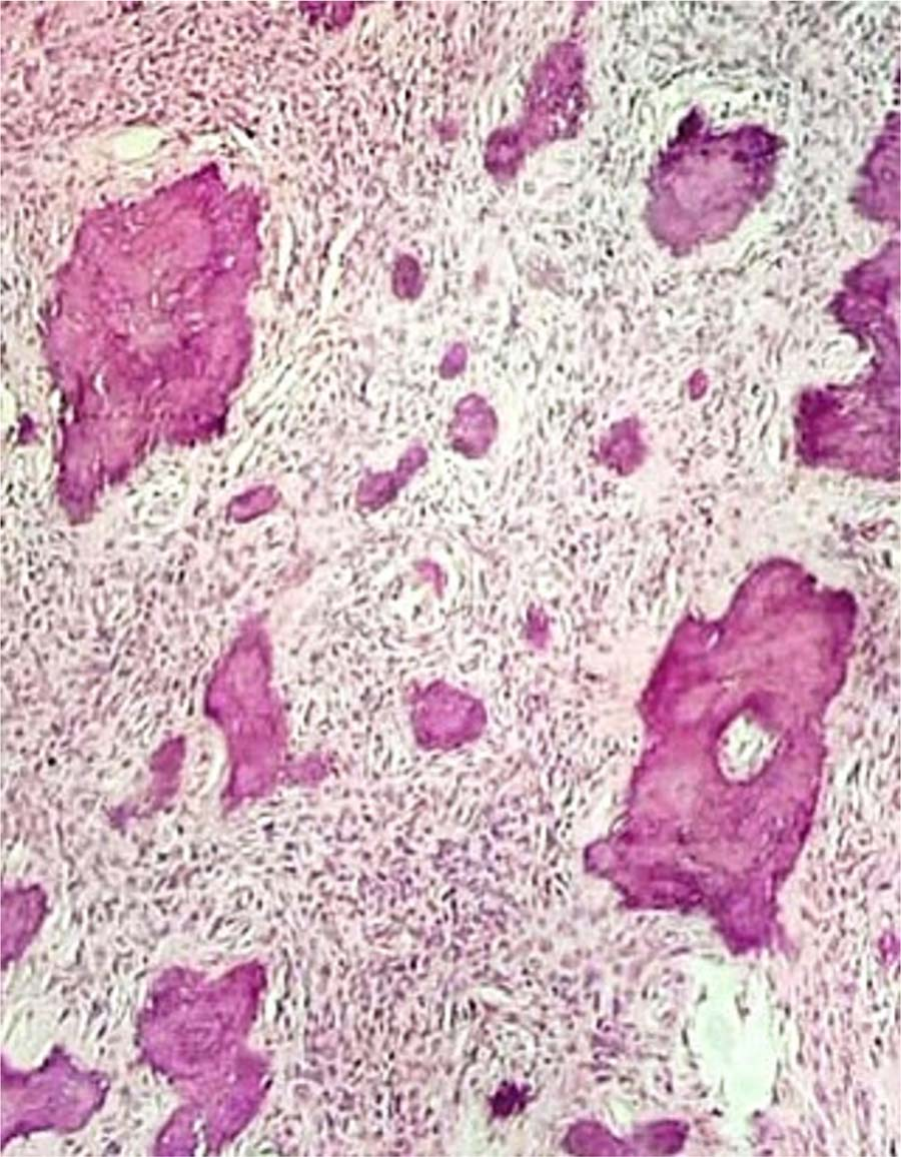
Histopathological findings. Low-power photomicrograph (H stain, ×10) showing cellular fibrous stroma with woven bone trabeculae.

Histopathological examination revealed cellular fibrous stroma with woven bone trabeculae showing osteoblastic rimming and a well-demarcated interface with adjacent bone, confirming conventional ossifying fibroma (Fig. 6) [12]. Resection margins were free of disease.

The postoperative course was uneventful with successful flap survival. The patient was monitored in the intensive care unit for the first 48 h with regular flap checks. She was started on a liquid diet on postoperative day 3 and gradually advanced to a soft diet. The surgical drain was removed when output was minimal.

**Figure 7:**
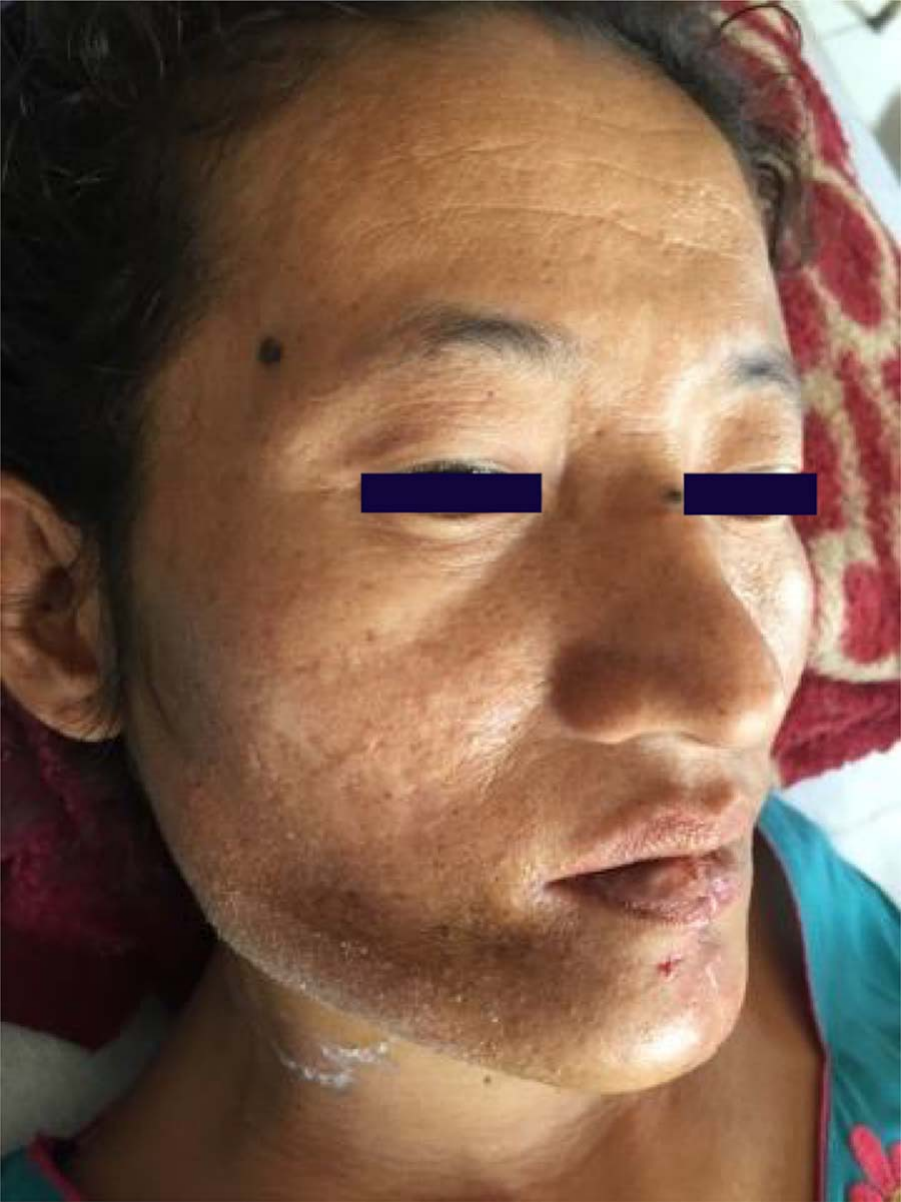
Postoperative day 10. Restored facial symmetry with minimal scarring following suture removal.

At discharge on postoperative day 10 following suture removal, clinical examination demonstrated excellent restoration of facial symmetry and mandibular contour with minimal, well-healed surgical scar (Fig. 7). The skin paddle showed healthy pink color with good capillary refill, confirming flap viability. The patient was tolerating a soft diet with no significant pain.

Unfortunately, as the patient came from a remote area and could not afford travel expenses, she did not return for follow-up after discharge. At the time of discharge, there was no evidence of complications, and the functional and esthetic outcomes were excellent.

## Discussion

This case demonstrates typical ossifying fibroma presentation in a young female with mandibular involvement [1, 3]. The radiographic features—well-defined margins, mixed radiodensity, cortical expansion without destruction—are characteristic [1, 4]. Segmental resection was appropriate given the extensive involvement, as resection minimizes recurrence compared to curettage for large lesions [1, 5].

Template modeling from the resected specimen allowed precise fibula contouring without the need for stereolithographic models or virtual surgical planning [8]. The free fibula flap offers adequate bone length (up to 25 cm), reliable vasculature, and excellent sculptability [6, 7, 9]. Microvascular anastomosis under loupe magnification achieves success rates of 97%–99%, comparable to the operating microscope, with advantages of reduced cost and setup time—particularly valuable in resource-limited settings [10, 11].

The excellent outcome at discharge with minimal scarring and restored facial contour demonstrates the effectiveness of this reconstructive approach [13]. This case also highlights challenges in providing complex surgical care to underserved populations, with the entire treatment provided pro bono. Loss to follow-up due to financial constraints represents a common barrier to comprehensive care in such settings.
